# Microfluidic Quantitative PCR Detection of 12 Transgenes from Horse Plasma for Gene Doping Control

**DOI:** 10.3390/genes11040457

**Published:** 2020-04-23

**Authors:** Teruaki Tozaki, Aoi Ohnuma, Mio Kikuchi, Taichiro Ishige, Hironaga Kakoi, Kei-ichi Hirota, Kanichi Kusano, Shun-ichi Nagata

**Affiliations:** 1 Genetic Analysis Department, Laboratory of Racing Chemistry, 1731-2 Tsurutamachi, Utsunomiya, Tochigi 320-0851, Japan; a-ohnuma@lrc.or.jp (A.O.); m-kikuchi@lrc.or.jp (M.K.); t-ishige@lrc.or.jp (T.I.); h-kakoi@lrc.or.jp (H.K.);; 2 Equine Department, Japan Racing Association, 6-11-1 Roppongi, Minato, Tokyo 106-8401, Japan; Kanichi_Kusano@jra.go.jp

**Keywords:** gene doping, horse, microfluidic qPCR, multiple-target detection, transgene

## Abstract

Gene doping, an activity which abuses and misuses gene therapy, is a major concern in sports and horseracing industries. Effective methods capable of detecting and monitoring gene doping are urgently needed. Although several PCR-based methods that detect transgenes have been developed, many of them focus only on a single transgene. However, numerous genes associated with athletic ability may be potential gene-doping material. Here, we developed a detection method that targets multiple transgenes. We targeted 12 genes that may be associated with athletic performance and designed two TaqMan probe/primer sets for each one. A panel of 24 assays was prepared and detected via a microfluidic quantitative PCR (MFQPCR) system using integrated fluidic circuits (IFCs). The limit of detection of the panel was 6.25 copy/μL. Amplification-specificity was validated using several concentrations of reference materials and animal genomic DNA, leading to specific detection. In addition, target-specific detection was successfully achieved in a horse administered 20 mg of the *EPO* transgene via MFQPCR. Therefore, MFQPCR may be considered a suitable method for multiple-target detection in gene-doping control. To our knowledge, this is the first application of microfluidic qPCR (MFQPCR) for gene-doping control in horseracing.

## 1. Introduction

Horses are being increasingly used in sports, with particular reference to activities such as horseracing, riding and polo. Careful management at various levels is required to ensure fair practice in sports. Horseracing has led to the selection of stallions and blood mares that can be used to produce the next generation of racehorses. Therefore, doping control is one important aspect [1]. Racing authorities and event organizers are responsible for doping control in horseracing, as well as in other equestrian sports. Prohibited substances mainly include low molecular weight compounds, such as β-agonists and steroids. These substances are detected via mass spectrometry and enzyme-linked immunosorbent assay (ELISA) [2,3]. The development of effective detection methods is expected to deter doping.

Recent advances in veterinary medical technologies have enabled gene therapy in horses, and several clinical studies have been reported [4,5,6]. Gene therapy may be technically categorized into gene transfer, gene silencing and gene editing.

Gene transfer is a technique that involves introducing a gene (generally referred to as a transgene) and expressing it in an organism to compensate for a functionally defective protein [7]. Gene silencing is a technique used for suppressing gene expression via mechanisms such as anti-sense oligonucleotides and RNA interference (RNAi) [8]. Gene editing artificially replaces, inserts or deletes a defective genomic sequence to cure genetic disorders or improve the body’s ability to fight diseases [9].

Although gene therapy contributes to veterinary medical care, the same technique may also be exploited for gene doping [10]. Therefore, in addition to conventional doping, the International Federation of Horseracing Authorities (IFHA) and Fédération Equestre Internationale (FEI) have prohibited gene doping—an activity that abuses or misuses gene therapy based on gene transfer, gene silencing and gene editing—in horseracing and equestrian sports.

A single study recently reported on detecting therapeutic nucleotides in gene-doping control [11], although many others have focused on transgene detection [12,13,14,15,16,17]. These detection methods are largely based on quantitative PCR (qPCR) techniques, such as real-time PCR or digital PCR. More recently, detection methods that use next-generation sequencers (NGS) have been developed [18]. NGS are highly sensitive for detecting single, targeted genes. However, several genes have been found to be involved in the athletic performance and growth traits of humans and animals. These genes are potential targets for gene doping. Thus, the development of detection methods capable of simultaneously testing many target genes assumed priority.

Microfluidic qPCR (MFQPCR), which performs PCR using integrated fluidic circuit (IFC) technology, is a recently developed technique [19]. It allows multiple targets from multiple samples to be simultaneously subjected to qPCR. The current study developed a microfluidic technology-based gene-doping detection method that enables the simultaneous analysis of transgenes for gene-doping purposes.

## 2. Materials and Methods 

### 2.1. Ethical Considerations

All animal experiments were approved by the Committee for Animal Research and Welfare of the Equine Research Institute (ERI), Japan Racing Association (JRA), as ethics protocol number: 18–23 (28 August 2018), and were conducted at facilities at the ERI, JRA, Shimotsuke, Japan.

### 2.2. Blood Collection and DNA Extraction

Blood collection from thoroughbred racehorses for method development and casework examples (1691 horses) was carried out at the ERI and the Miho Training Center, JRA, by veterinarians, while taking care not to stress the horses. Blood samples were obtained at resting conditions in their respective stables. Horse blood was collected into BD Vacutainer^®^ spray-coated K2EDTA tubes (Becton Dickinson Company, Franklin Lakes, NJ, USA), and plasma was separated via centrifugation at 1500× *g* for 10 min. Separated plasma was stored at −40 °C.

DNA was extracted from 1 mL of plasma via a Prepito Circulating NA 1k Kit (PerkinElmer, Waltham, MA, USA) using a Prepito-D (Perkin Elmer) instrument. The extract was dissolved with Milli-Q water to obtain a final volume of 90 μL.

### 2.3. Animal Genomic DNA Preparations

Horse genomic DNA was extracted from whole blood using a DNeasy Blood & Tissue Kit (Qiagen, Hilden, Germany). Genomic DNA of human, monkey, dog, cat, bovine, goat, sheep, porcine, mini-pig, camel, llama, mouse, rabbit, chicken and donkey were purchased from Zyagen (San Diego, CA, USA). Extracted or obtained genomic DNA was diluted to 20 ng/μL in Milli-Q water.

### 2.4. Preparation of Cloned Horse Transgenes as Reference Material

Based on the genomic information (EquCab2.0, GCA-000002305.1), 12 horse transgenes, creatine kinase, muscle (CKM), erythropoietin (EPO), fibroblast growth factor 2 (FGF2), follistatin (FST), growth hormone 1 (GH1), insulin like growth factor 1 (IGF1), myostatin (MSTN), phosphoenolpyruvate carboxykinase 1 (PCK1), pyruvate dehydrogenase kinase 4 (PDK4), peroxisome proliferator activated receptor delta (PPARD), vascular endothelial growth factor (VEGF) and zinc finger and AT-hook domain containing (ZFAT), containing open reading frames (ORFs) and untranslated regions were synthesized (Fasmac, Kanagawa, Japan) (Table 1). Synthesized sequences were cloned into the plasmid vector, pUCFa (r-Amp^+^, ColE1_ori^+^); transformed into JM109 competent cells (Takara Bio Inc., Kusatsu, Shiga, Japan) and cultured in LB medium (Amp^+^). The plasmids were purified using a Wizard Plus SV Minipreps DNA Purification System (Promega, Madison, WI, USA) and dissolved in Milli-Q water to a concentration of approximately 10 ng/μL by NanoDrop One^C^ (Thermo Fisher Scientific, Waltham, MA, USA), which was used to initially measure concentrations. Sequences of the cloned transgenes were confirmed via Sanger sequencing.

In addition, the *EPO* transgene administered to horses in this study was prepared according to a method in our previous study [16]. Briefly, a 579-bp horse *EPO* containing ORF (192 amino acids) and a termination codon was cloned into a plasmid vector pBApo-CMV (r-Amp^+^, CMV IE promoter, HSV TK poly A; Takara Bio). A large-scale (20 mg) preparation was performed by Takara Bio Inc. The purified vector was filtered through a 0.22-μm filter to remove endotoxins, and the sequence was confirmed by Sanger sequencing.

### 2.5. Quantitation of Transgenes Cloned into Plasmid Vector

The 12 transgenes dissolved in Milli-Q water were quantified using a NanoDrop One^C^, Qubit dsDNA HS Assay Kit (Thermo Fisher Scientific) and droplet digital PCR (ddPCR; Bio-Rad, Hercules, CA, USA) using a TaqMan probe. For ddPCR, we followed a protocol used in our previous study [16]. Finally, values quantified by ddPCR were used for concentrations of reference material (RM) in the current study.

### 2.6. Designing Primers and Probes for MFQPCR Detection

**Figure 1:**
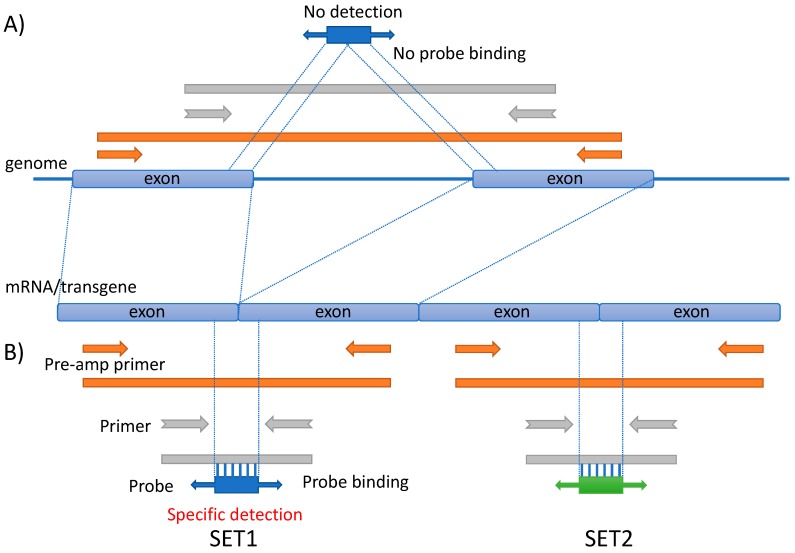
Probe and primer design for microfluidic quantitative PCR (MFQPCR) detection. Two assays (SET1 and SET2) were designed for each transgene. Forward and reverse primers for MFQPCR were designed to target different exons. TaqMan probe for MFQPCR was designed to target exon/exon junctions. Forward and reverse primers for pre-amplification were designed to include the forward and reverse primers for MFQPCR. While forward and reverse primers for pre-amplification and MFQPCR may amplify PCR products, including genomic DNA regions, TaqMan probes do not anneal to the PCR product, including genomic DNA regions (**A**). TaqMan probes specifically anneal to PCR products having exon/exon junction sequences (**B**).

An image of probe and primer design for microfluidic quantitative PCR (MFQPCR) detection is shown (Figure 1). Two sets, namely SET1 and SET2, were prepared for each one and were targeted to different regions (exon/exon junction). In order to detect transgenes, TaqMan-MGB probes and primers (forward and reverse), as well as pre-amplification primers (pre-forward and pre-reverse), were designed and synthesized (Thermo Fisher Scientific K.K., Minato, Tokyo, Japan). Each primer (forward and reverse) was designed to target different exons, and the probe was designed to target the junction of both exons. Pre-amplification primers were designed on regions that included the TaqMan-probe and the primers. Designed probes and primers have not been listed for the purposes of actual gene-doping tests. Sequence information of the probes and primers will be provided by a confidentiality agreement with the corresponding author.

### 2.7. Preparation and Quantification of Control Samples

Positive control samples (PCSs) of the 12 transgenes were prepared. Each RM of the 12 transgenes was serially diluted as follows: SD1 (1/10 of RM), SD2 (1/10 of SD1), SD3 (1/10 of SD2), SD4 (1/10 of SD3), SD5 (1/10 of SD4), SD5.5 (1/2 of SD5), SD6.5 (1/10 of SD5.5) and SD7.5 (1/10 of SD6.5). Copy concentrations (copy/μL) for each SD5 of the 12 transgenes were measured using ddPCR. Theoretical copy concentrations of SD3 were calculated based on measured copy concentrations of SD5, within the upper limit of quantification. From the theoretical copy concentrations of SD3, we constructed a PCS consisting of a mixture of the 12 transgenes, using equivalent copy numbers of all 12 transgenes. Next, the PCS was diluted to 10,000, 1000, 100, 10, 5.0 and 2.5 copies/μL, which were named PCS_10000, PCS_1000, PCS_100, PCS_10, PCS_5.0 and PCS_2.5, respectively. A 10-ng/μL horse genomic DNA solution was used for all dilutions. Two negative control samples (NCSs)—10 ng/μL of horse genomic DNA solution and Milli-Q water—were used for all experiments in this study.

### 2.8. MFQPCR Detection Using Dynamic Array Integrated Fluid Circuits (IFCs)

Prior to MFQPCR, the purified DNA, PCSs and NCSs were pre-amplified using SET1 and SET2 pre-amp primer pools, respectively. Briefly, the two separate assay pools, each containing 12 primer pairs, were prepared by mixing forward and reverse pre-amp primers and diluting with TE buffer (Thermo Fisher Scientific) at a final concentration of 180 nM of each primer. PCR reaction was carried out using 5-μL primer mix, including 1.25-μL template DNA, 1-μL PreAmp Master Mix (Fluidigm Corporation, South San Francisco, CA, USA) and 1.25-μL pooled assay mix. The thermal profile of the pre-amplification consisted of incubation at 95 °C for 2 min followed by 19 cycles of 95 °C for 15 s and 60 °C for 4 min by GeneAmp PCR System 9700 (Thermo Fisher Scientific).

The pre-amplified products (5 μL) were diluted by adding 20 μL of TE buffer and stored at −20 °C until needed for further analyses. MFQPCR was carried out with 192.24 Dynamic Array IFC for Gene Expression (Fluidigm) for transgene detection. Sample mix and 10× assay mix were prepared according to the manufacturer’s instructions. For the sample mix, 1.8 μL of diluted pre-amplified PCR products were combined with 2.0 μL of TaqMan Fast Universal PCR Master Mix (2×), No AmpErase UNG (Thermo Fisher Scientific) and 0.2 μL of 20× GE Sample Loading Reagent (Fluidigm). For 10× assay mix, stock solutions of 20× primer-probe mix (18 pmol/μL of primers and 5 pmol/μL of probe) were prepared for each 24 assays. The 10× assay mix were then prepared using 2 μL of the 20× primer-probe mix with 2 μL of the 2× Assay Loading Reagent (Fluidigm). The sample and the 10× assay mixes were pipetted onto appropriate inlets on 192.24 Dynamic Array IFC, primed and loaded into microfluid lines with Juno System (Fluidigm). Next, MFQPCR was performed on the Biomark HD System (Fluidigm) using a fast program that consisted of an incubation step at 95 °C for 60 s followed by 35 cycles of 96 °C for 5 s and 60 °C for 20 s.

MFQPCR was also carried out with 48.48 Dynamic Array IFC for transgene detection of several samples through similar procedures. In this case, the two assays (each 12-detection) were carried out in duplicates at the same IFCs.

Fluorescence emission was recorded after each cycling step. All qPCR operations and data analyses were conducted using Biomark Data Collection Software and Real-Time PCR Analysis Software (Fluidigm), respectively.

### 2.9. Administration of EPO Transgene

The number of animals used was kept to a minimum in this case study in order to comply with animal ethics and welfare guidelines. Administration of the *EPO* transgene to a horse has been previously performed by us [16]. Briefly, a thoroughbred horse (male, 11 years old, 465 kg) at the ERI, JRA was intramuscularly injected with 20 mg of the horse *EPO* transgene, and blood samples were collected as follows: before administration, 15 min, 3 h, 6 h, 12 h, 24 h, 2 d, 3 d, 4 d, 5 d, 6 d, 7 d, 14 d, 21 d and 28 d after administration. Collected blood was centrifuged, and the plasma was separated and stored at −40 °C.

## 3. Results and Discussion

### 3.1. Targeted Genes for Gene-Doping Control

The present study targeted *CKM*, *EPO*, *FGF2*, *FST*, *GH1*, *IGF1*, *MSTN*, *PCK1*, *PDK4*, *PPARD*, *VEGF* and *ZFAT* for the control of gene doping in horseracing. CKM, PCK1, PDK4 and PPARD are associated with energy transduction, gluconeogenesis and glucose and fatty acid metabolism, respectively [20,21,22,23]. GH1 and IGF1 encode the central elements of a key pathway influencing growth in animals [24]. EPO is a hematopoietic factor that plays an important role in erythropoiesis [25]. MSTN is a negative regulator of muscle growth, and FST inhibits MSTN activity by binding to MSTN [26,27]. ZFAT is associated with body weight in thoroughbred horses [28]. FGF2 and VEGF are also growth factors and therefore used for treating tendons of horses via gene therapy [4]. In addition, some of these genes may directly or indirectly affect the racing performance of thoroughbred horses [29,30,31,32]. Therefore, these 12 genes were chosen as the initial, minimum targets for gene-doping control in horseracing and detected in a panel of 24 assays, SET1 and SET2, using MFQPCR.

### 3.2. Quantitation of Reference Materials (RMs)

**Figure 2:**
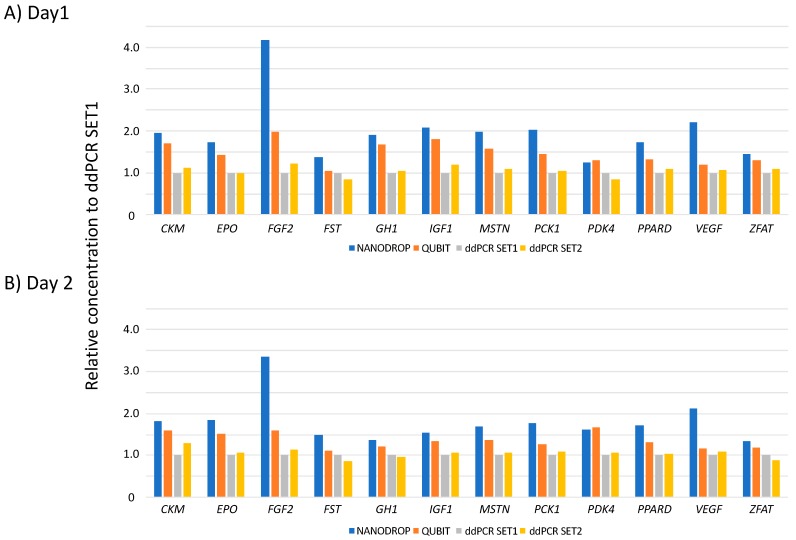
Relative concentrations of reference materials (RMs) among the NanoDrop One^C^, Qubit dsDNA HS Assay and ddPCR using SET1 and SET2. Concentrations were measured twice at days 1 (**A**) and 2 (**B**). Values of NanoDrop, Qubit and droplet digital PCR (ddPCR) SET2 were calculated for a SET 1 ddPCR value of 1.

Reference materials (RMs) of 12 transgenes were quantified via NanoDrop One^C^, Qubit dsDNA HS Assay and ddPCR using SET1 and SET2. The weight-based concentrations (ng/μL) on NanoDrop One^C^ and Qubit dsDNA HS assays were converted to copy concentrations (copy/μL) based on the molecular weights of these transgenes. This experiment was repeated twice on different days (Day1 and Day2). Relative values of copy concentrations obtained using different quantification methods when the concentration of ddPCR using SET1 was 1 are shown in Figure 2. Relative ddPCR values obtained using SET2 ranged from 0.84 (*PDK4*) to 1.22 (*FGF2*) on Day1 and 0.86 (*FST*) to 1.29 (*CKM*) on Day2. The difference between SET1 and SET2 was not significant.

However, relative values obtained by NanoDrop One^C^ ranged from 1.24 (*PDK4*) to 4.17 (*FGF2*) on Day1 and 1.33 (*ZFAT*) to 3.35 (*FGF2*) on Day2. Relative values obtained via Qubit dsDNA HS Assay ranged from 1.24 (*PDK4*) to 1.97 (*FGF2*) on Day1 and 1.12 (*FST*) to 1.68 (*PDK4*) on Day2. Concentrations obtained by NanoDrop One^C^ and Qubit dsDNA HS Assay were higher than those obtained via ddPCR.

*Escherichia coli*-derived genomic double-stranded and single-stranded nucleotides, such as DNA and RNA, may be present in purified plasmid solutions, and it is difficult to remove these nucleotides completely via the purification process, suggesting that concentrations measured by NanoDrop One^C^ and Qubit dsDNA HS assay may be higher than the actual concentrations. Therefore, ddPCR was selected as the quantification method of RMs for gene-doping detection in subsequent experiments.

### 3.3. Limit of Detection (LOD) of MFQPCR

**Figure 3:**
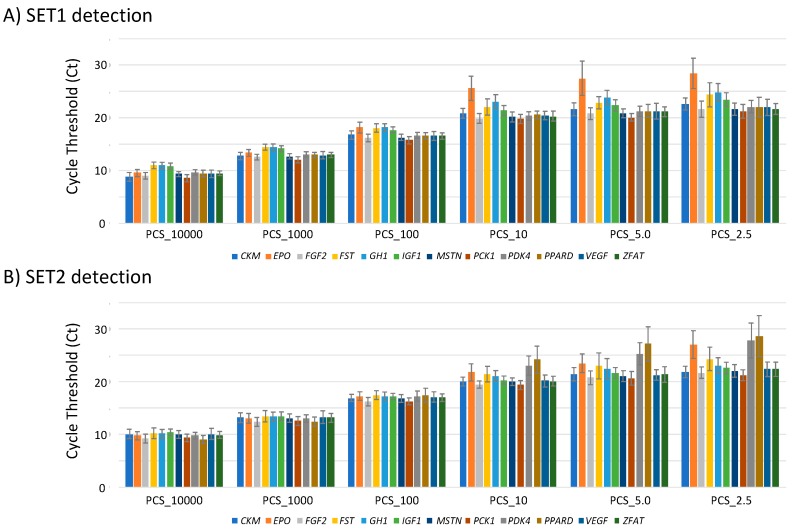
Cycle threshold (Ct) of each transgene on positive control sample (PCS)_10000 (12,500 copies), PCS_1000 (1250 copies), PCS_100 (125 copies), PCS_10 (12.5 copies), PCS_5.0 (6.25 copies) and PCS_2.5 (3.125 copies) based on SET1 (**A**) and SET2 (**B**) detections. The experiment was repeated 35 times, and the mean values are shown.

Using PCS_10000, PCS_1000, PCS_100, PCS_10, PCS_5.0 and PCS_2.5 as templates, MFQPCR detection for 24 assays was repeated 35 times. Distributions of the cycle threshold (Ct) for each PCS obtained via the Biomark HD System are shown (Figure 3). While all MFQPCR detections using PCS_10000, PCS_1000 and PCS_100 as templates were successful, some, such as those using PCS_10, PCS_5.0 and PCS_2.5, did not show any amplification. In 840 detections (24 assays × 35 experiments), 1 (0.12%), 9 (1.07%) and 70 (8.33%) nonamplifications were observed in PCS_10, PCS_5.0 and PCS_2.5, respectively, suggesting that PCS_5.0 (successful detection rate > 95%) could be used as the limit of detection (LOD). Since 1.25 μL was used as the initial sample volume of the template, 6.25 copies (5.0 copy/μL × 1.25 μL) were considered as the LOD for MFQPCR detection in this study. Nonamplification may have been due to the effective quantity of copies that could not reach each dilution aliquot because of the possible overestimation of the RM values by ddPCR.

### 3.4. MFQPCR-Amplification Specificity

**Figure 4:**
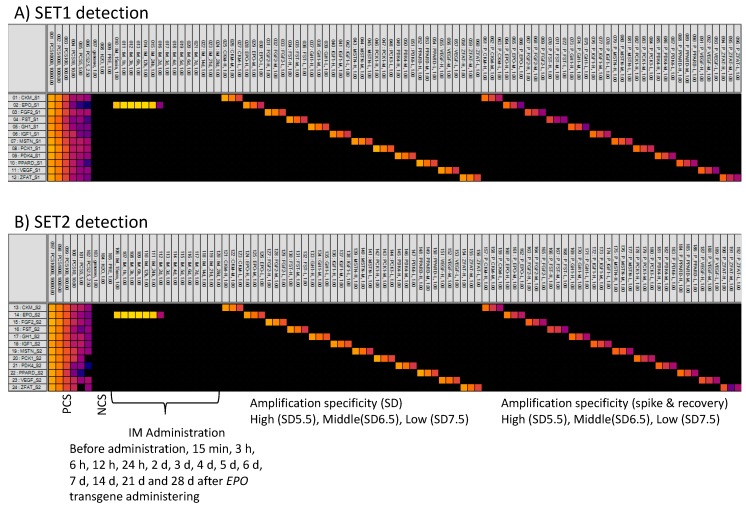
Image of microfluidic quantitative PCR (MFQPCR) detection. Positive control samples (PCS_10000, PCS_1000, PCS_100, PCS_10, PCS_5.0 and PCS_2.5); negative control samples (horse genomic DNA and Milli-Q) and plasma at: before administration, 15 min, 3 h, 6 h, 12 h, 24 h, 2 d, 3 d, 4 d, 5 d, 6 d, 7 d, 14 d, 21 d and 28 d after *EPO* transgene administering high, middle and low concentration of PCSs for each transgene, and spiked and recovered samples for each transgene were MFQPCR-amplified using SET1 (**A**) and SET2 (**B**) detections. Vertical axis is assays; CKM, EPO, FGF2, FST, GH1, IGF1, MSTN, PCK1, PDK4, PPARD, VEGF and ZFAT for SET1 (**A**) and SET2 (**B**) detections. Light yellow and orange indicate lower cycle thresholds (Cts) (high copy concentrations). Dark purple depicts high Cts (low copy concentrations). Black indicates nonamplification. NCS: negative control sample.

Amplification specificity was evaluated using SD5.5 (high concentration), SD6.5 (middle concentration), SD7.5 (low concentration) and extracts from 1-mL samples of plasma that were spiked with 10 μL of SD5.5, SD6.5 or SD7.5. All SET1 and SET2 assays detected their targeted transgenes only (Figure 4). Neither contamination nor interference was observed during our MFQPCR detection, indicating that MFQPCR enabled specific qPCR detection of multiple targets in multiple samples.

Human, monkey, dog, cat, bovine, goat, sheep, porcine, mini-pig, camel, llama, mouse, rabbit, chicken, donkey and horse genomic DNA (10 ng) was used to test the detection capability of MFQPCR, performed in triplicates of each sample. All genomes were not amplified by the 24 assays, except for the following eight: sheep genome by EPO_SET1 (Ct = (24.7)), bovine genome by VEGF_SET1 (Ct = (22.9)) and VEGF_SET2 (Ct = (23.7)), llama genome by VEGF_SET2 (Ct = (23.0) and (21.9)), mouse genome by VEGF_SET2 (Ct = (22.8) and (22.7)) and rabbit genome by VEGF_SET2 (Ct = (28.0)). While the genomes were amplified once or twice, no genomes were amplified in all three replicates. The Cts of almost all the amplified assays were higher than the Ct of the LOD determined in this study.

This demonstrated that the primers and probes used for MFQPCR were specific for the amplification of the 12 horse transgenes.

### 3.5. Detection of the EPO Transgene After It Was Administered to a Horse

A thoroughbred was administered 20 mg of the *EPO* transgene, and its blood was sampled to an EDTA collection tube using the following schedule: before administration, 15 min, 3 h, 6 h, 12 h, 24 h, 2 d, 3 d, 4 d, 5 d, 6 d, 7 d, 14 d, 21 d and 28 d after administration. Blood was centrifuged immediately after collection, and the separated plasma was stored at −40 °C. Next, the *EPO* transgene was extracted from 1 mL of plasma.

The assays detected the *EPO* transgene only at 15 min, 3 h, 6 h, 12 h, 24 h and 2 d following its administration (Figure 4), while the other 11 transgenes in the collected horse plasma were not detected by MFQPCR. The plasma showed signals indicating high concentrations of the *EPO* transgene at 15 min, 3 h, 6 h, 12 h and 24 h following its administration (Figure 4; yellow signal) and signals indicating low concentrations 2 d after administration (Figure 4; purple signal). These concentration trends were similar to those seen in one of our previous studies [16], in which ddPCR was used to detect the *EPO* transgene. All the signals from 15 min to 2 d were over the LOD (6.25 copy/μL).

Therefore, the series of methods using the MFQPCR technique developed in this study expectedly enabled target-specific transgene detection in horses administered transgenes that show potential as gene-doping material. However, the data should be interpreted with caution, because it was only one horse which produced these data.

### 3.6. Casework Example

The detection of 12 transgenes, which showed potential as gene-doping material, was performed using 1.25-μL extracts (mean: 8.4 ng/μL, standard deviation: 10.4) obtained from the plasma of 1691 thoroughbred racehorses. MFQPCR products amplified via both SET1 and SET2 were not observed at copy numbers exceeding the LOD (6.25 copies/reaction tube), except for some samples in SET1 (one case, VEGF_SET1), as well as in SET2 (four cases, FGF2_SET2, MSTN_SET2, PCK1_SET2 and PDK4_SET2), which showed an amplification signal indicative of a low copy number. Although some assays showed nonspecific amplification signals, no transgenes were detected in both assays (SET1 and SET2), possibly because the racehorses sampled in this study had not been administered any of the 12 transgenes. To counter difficulties associated with completely preventing nonamplification, at least two assays per targeted transgene should be considered in order to verify certain cases of doping.

Furthermore, as it may be difficult to completely prevent nonspecific amplification, it would be more appropriate to define a detection threshold that protects against false positive results, particularly in cases showing low copy numbers. The results of this study indicated that 6.25 copies/reaction tube would be a suitable detection threshold for MFQPCR screening.

## 4. Conclusions

To the best of our knowledge, the method described here represents the first application of microfluidic qPCR (MFQPCR) for the purpose of controlling gene doping in horseracing. Currently, over 20,000 genes are annotated in the horse genome, allowing many genes to be targeted as transgenes for the express purpose of gene doping [33]. Although a small number of causative genes encoding traits associated with racing performance and growth have been identified in horses [29,30,31,32], multiple candidate genes encoding athletic and growth traits have been already identified in humans and/or animals [34]. Therefore, many target genes may need to be screened for the purposes of gene-doping control. While 192 × 24 IFCs were used to detect 12 genes using 24 assays in this study, 48 × 48 and 96 × 96 IFCs could also be purchased from the manufacturers, indicating that a maximum of 48 or 96 assays could be made available for each individual experiment.

In addition, MFQPCR uses a microfluidic device that requires only small amounts of reagents, compared to conventional qPCR using 96-well plates. This indicates that detection via MFQPCR is cost-effective. Thus, MFQPCR appears to be especially suitable for screening gene doping. Although the current method used 24 assays to detect 12 genes in order to provide an example of gene-doping detection, it may be easily adapted to detect other target genes by designing new primers and probes.

The current study evaluated a casework example of 1691 thoroughbred racehorses. Almost all these horses were found negative of gene doping; however, in some cases, signals representative of low copy numbers were rarely detected via either SET1 or SET2. This may have been due to nonspecific amplification. Thus, confirmation experiments should follow such screenings. Our previous study [16] developed and evaluated a gene-doping detection method using ddPCR, which is able to quantify gene-doping materials. Therefore, a combination of MFQPCR for screening and ddPCR for confirmation may be more suitable for the detection of gene doping under certain circumstances.

## Figures and Tables

**Table 1:** Genes targeted for gene-doping control in horseracing.

Gene Name	Symbol	Function or Related Traits
creatine kinase, muscle	*CKM*	energy transduction
erythropoietin	*EPO*	hematopoiesis
fibroblast growth factor 2	*FGF2*	cell growth, morphogenesis, tissue repair
follistatin	*FST*	muscle growth (antagonist of MSTN)
growth hormone 1	*GH1*	growth control
insulin like growth factor 1	*IGF1*	systemic body growth stimulation
myostatin	*MSTN*	muscle growth (negative regulator)
phosphoenolpyruvate carboxykinase 1	*PCK1*	regulation of gluconeogenesis
pyruvate dehydrogenase kinase 4	*PDK4*	regulation of glucose and fatty acid metabolism
peroxisome proliferator activated receptor delta	*PPARD*	oxidative (fat-burning) capacity
vascular endothelial growth factor	*VEGF*	vasculogenesis, angiogenesis
zinc finger and AT-hook domain containing	*ZFAT*	effect on body weight in thoroughbreds
